# Successful mechanical thrombectomy in acute bilateral M1 middle cerebral artery occlusion: a case report and literature review

**DOI:** 10.1186/s12883-023-03173-y

**Published:** 2023-03-24

**Authors:** Zhiqiang Li, Shuhui Wu, Shuzhi Zhao, Ning Li, Weibin Ma, Guisheng Jiang, Lingling Liu, Guoxian Jing

**Affiliations:** 1 Department of Neurology, Liaocheng People’s Hospital, NO. 67, West Dongchang Road, Shandong, Liaocheng City, 252000 China; 2 Department of Traditional Chinese Medicine, Liaocheng Third People’s Hospital, No.62, Weiyu Road, Shandong, Liaocheng City, 252000 China; 3 Department of Neurology, Liaocheng Third People’s Hospital, No.62, Weiyu Road, Shandong, Liaocheng City, 252000 China

**Keywords:** Bilateral MCA stroke, Endovascular treatment, Ischemic stroke, Intravenous thrombolysis, Bridge therapy

## Abstract

**Background:**

Acute bilateral occlusion of the middle cerebral artery (MCA) is a very rare condition, and most cases are accompanied by a poor prognosis. However, mechanical thrombectomy (MT) for bilateral MCA is challenging. Here, we report a case of acute unilateral MCA occlusion with sequential acute occlusion of the bilateral MCA during intravenous thrombolysis (IVT). We urgently performed bilateral MT of the MCA and effective recanalization.

**Case presentation:**

The patient is a 73-year-old man who complained of a sudden adverse influence on speech and an inability to move his left limb for 2 h. He had a history of paroxysmal atrial fibrillation, but had never used any anticoagulants before. Head and neck computed tomography angiography (CTA) showed embolism in the right M1 MCA. During intravenous alteplase thrombolytic therapy, the patient suddenly became unconscious. Cerebral angiography showed occlusion of the M1 segment of the bilateral MCA in the patients. MT of the bilateral MCA was performed using a combination of a stent retriever and an aspiration catheter with mTici 3 revascularization. On the second day, the patient became conscious, although he had remaining symptoms of speech insufficiency and weakness of the left limb. The mRS score was 2 90 days after the operation.

**Conclusions:**

Acute bilateral occlusion of the M1 segment of the MCA is extremely rare and is accompanied by high morbidity and high mortality. Intravenous alteplase thrombolysis can increase the risk of atrial thrombus shedding in patients with atrial fibrillation, so patients with acute bilateral MCA occlusion in the M1 segment chose direct MT or bridging therapy, which remains controversial, and the sequence of MT remains to be discussed. Nevertheless, early endovascular treatment can decrease the morbidity and mortality of such patients.

## Background

IVT is recommended as an important treatment for acute cerebral infarction in many guidelines [1]. However, the causes of acute cerebral infarction in patients with atrial fibrillation are mainly large vascular occlusion, and the efficacy of IVT is inconsistent. In addition, intravenous thrombolytic therapy may also cause atrial thrombosis in patients with atrial fibrillation and lead to large vascular embolism [2]. MT has been demonstrated to be useful for the treatment of ischemic stroke in patients with large vessel occlusions [3, 4]. However, cerebral infarction caused by acute bilateral occlusion of the M1 segment of the MCA is rare, and acute MT has become an important strategy for its treatment. Therefore, the choice of bridging therapy or direct MT for acute cerebral infarction caused by large vascular embolization remains controversial [5, 6]. In this paper, we report a case of acute right MCA occlusion with sequential acute occlusion of the bilateral MCA during IVT. We urgently performed bilateral MT of the MCA and successful recanalization.

## Case presentation

**Fig. 1:**
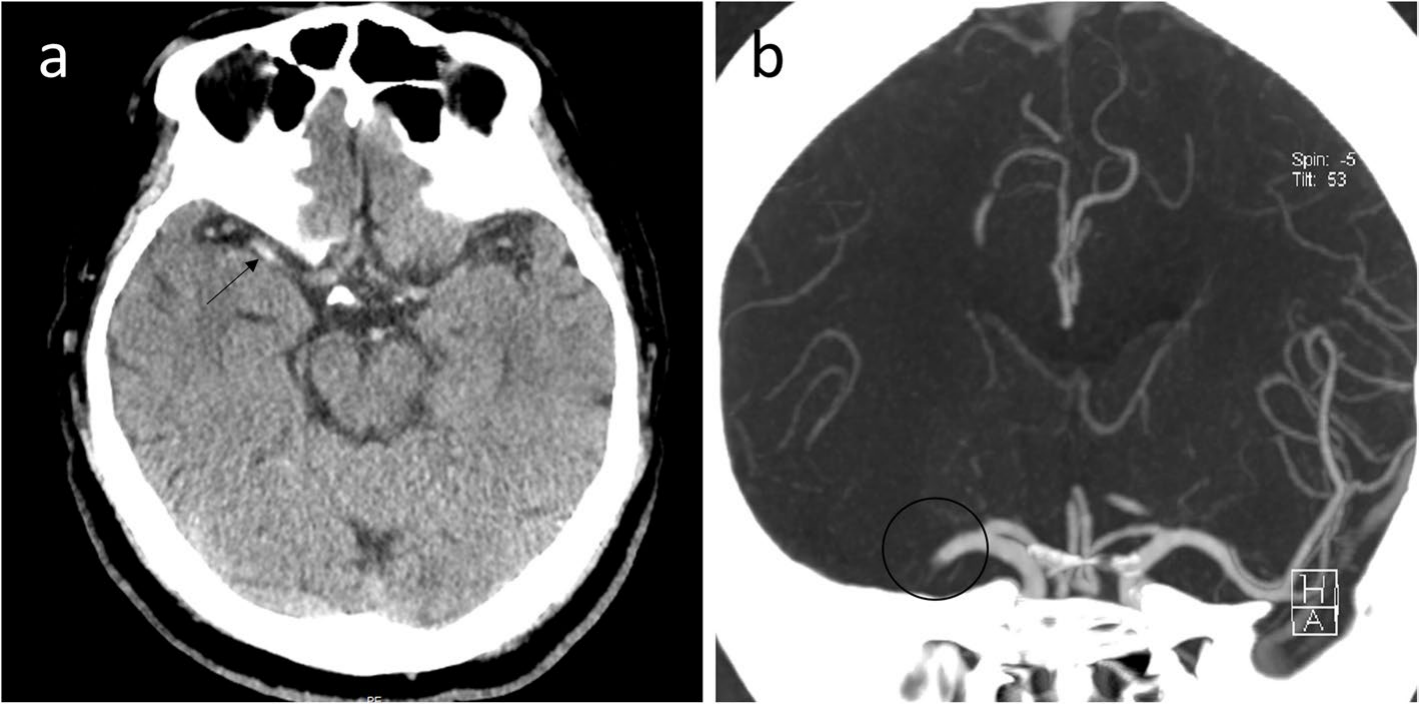
Preoperative imaging examination. **a** Brain computed tomography showed a high density of the right MCA (black arrow). **b** Computed tomography angiography (CTA) showed a right M1 occlusion (black circle)

The patient is a 73-year-old man who complained of sudden incomprehensible speech and the inability to move his left limb for 2 h. He suffered paroxysmal atrial fibrillation but never used anticoagulants. Neurological examination of the patient indicated motor aphasia, left central facial paralysis, grade 1 left limb muscle strength, a positive pathological sign of the left, and shallow sensory loss of the left limb. The initial National Institutes of Health Stroke Scale (NIHSS) score was 15. Brain computed tomography (CT) showed a high density sign of the right MCA (Fig. 1a). Alberta stroke program early CT score (ASPECTS) was 2. Head and neck computed tomography angiography (CTA) showed embolisms in the right M1 MCA (Fig. 1b), and electrocardiographic findings were normal. At 109 min after onset, the patient was given IVT with alteplase at a dose of 0.9 mg / kg. We urgently reviewed the brain CT to exclude off secondary cerebral bleeding after the patient became unconscious during IVT with a Glasgow coma score of 5.

**Fig. 2:**
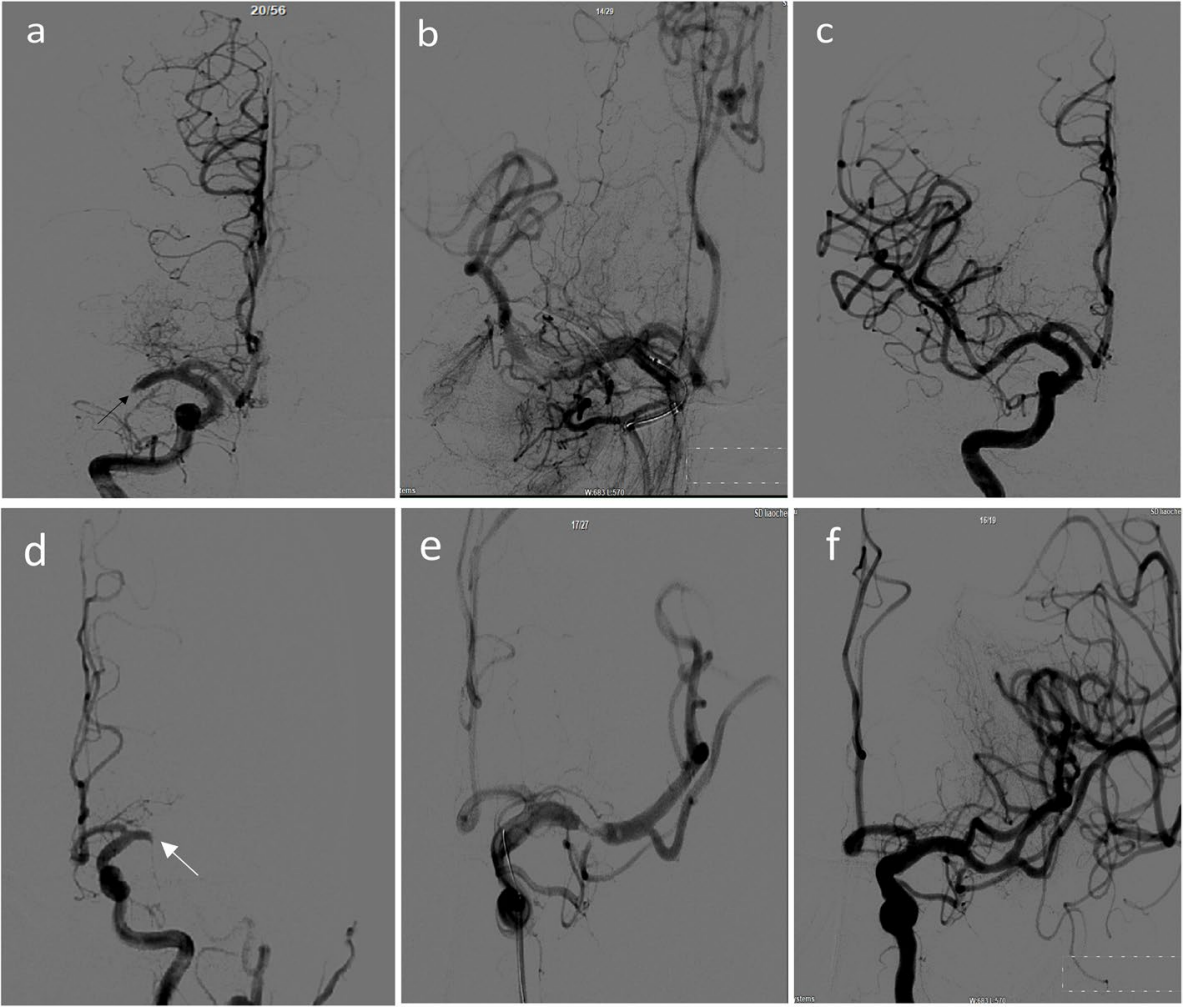
MT of bilateral M1 occlusion using the Solitaire FR device. (EV3, California, USA) and a Navien catheter (EV3, Irvine, USA). **a** Right M1 occlusion (black arrow). **b** One trunk of the right MCA is unobstructed, but the other is not visible. **c** Right MCA TICI 3 revascularization. **d** Left M1 occlusion (white arrow). **e** One trunk of the right MCA is unobstructed, but the other is not visible. **f** Left MCA TICI 3 revascularization

MT was performed 205 min after onset. An 8F sheath was inserted into the right femoral artery of the patient, and cerebral angiography showed bilateral occlusion of the MCA in the M1 segment (Fig. 2a, d). A 5F 125-cm Navien (EV3, Irvine, USA) was placed at the end of the right internal carotid artery with a 90 cm Max Neuron (Penumbra, Alameda, USA) sheath. Through the Navien catheter, a Synchro2 microguide (Stryker, Fremont, USA) and Rebar-18 microguide (EV3, Irvine, USA) were carefully placed in the M3 segment of the right MCA through the M1 occlusion segment of the right MCA. Subsequently, angiography confirmed the occlusion in the true lumen. Solitaire FR (6 mm × 30 mm) (EV3, California, USA) was placed in the occluded segment and the right MCA was unobstructed. After 10 min of observation, the stent was removed and Navien catheter aspiration was performed. A large amount of thrombosis was removed. Angiography showed that the blood flow of the main branch of the right MCA was not obstructed with mTici grade 3 (Fig. 2c). The same method was then used to remove left MCA M1 thrombosis. Angiography showed recanalization of the trunk and branches of the left MCA with mTici grade 3 (Fig. 2f).

**Fig. 3:**
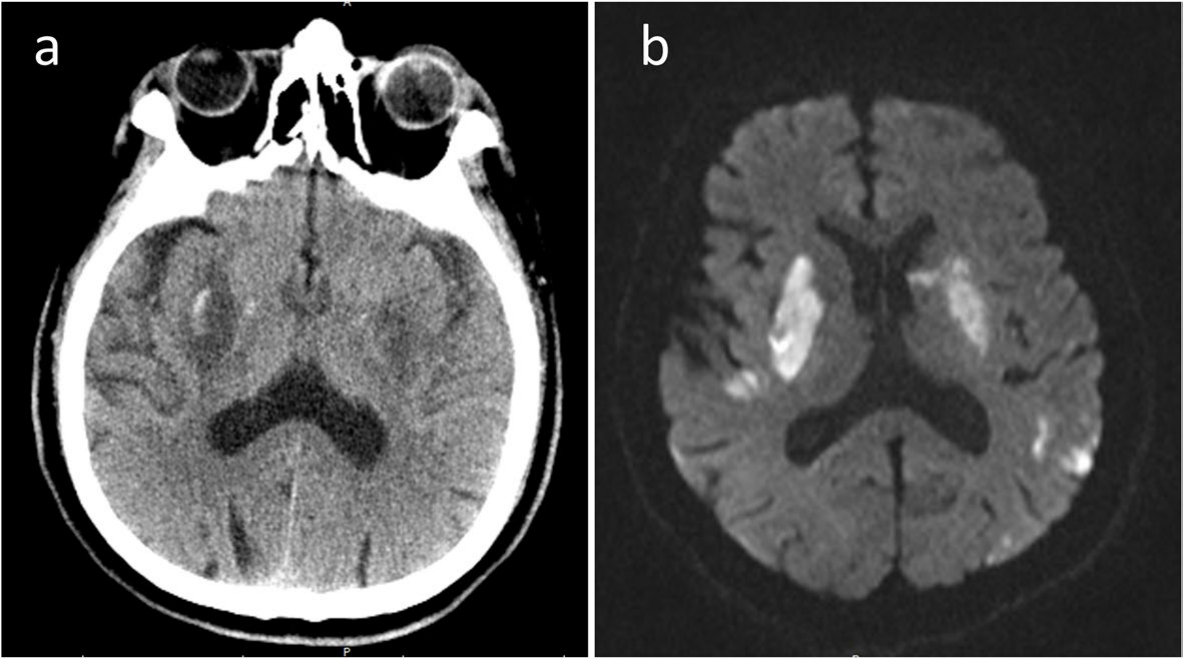
Postoperative imaging examination. **a** Brain CT showing a small amount of bleeding in the right basal ganglia. **b** Diffusion-weighted magnetic resonance axial imaging showing new cerebral infarction in the bilateral basal ganglia and bilateral cortex

**Fig. 4:**
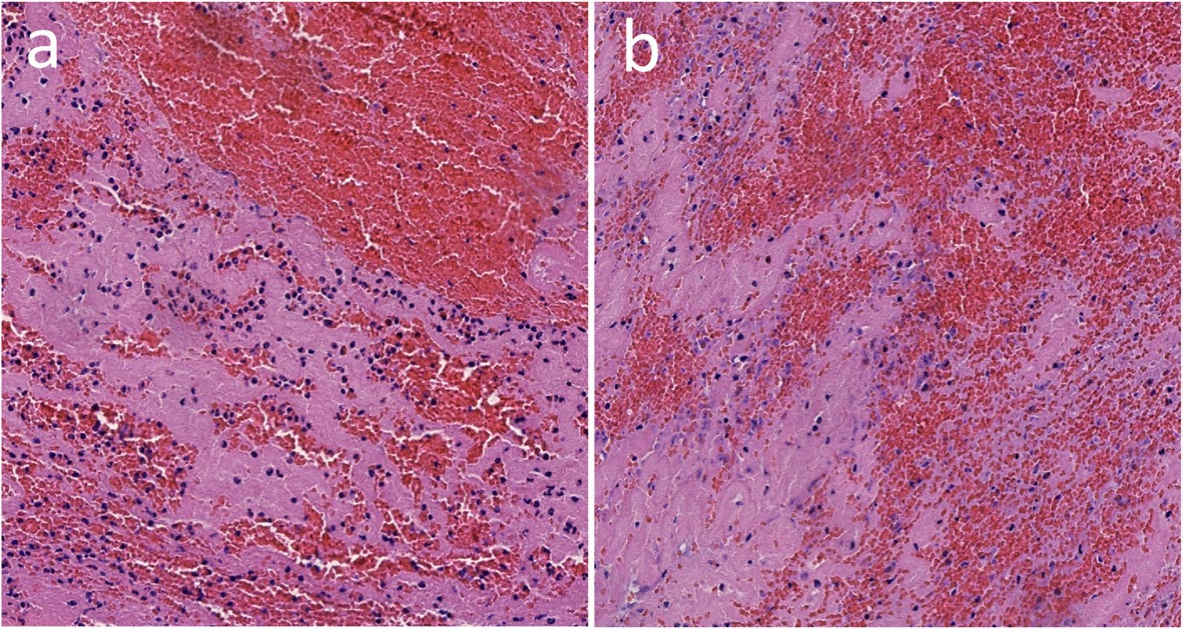
Images of the pathological results of the bilateral embolus. **a** Pathological results of left thrombus. **b** Pathological results of right thrombus. Mixed thrombosis containing fibrinous infiltration, red blood cell infiltration and inflammatory cell infiltration

On the second day after the operation, the patient regained consciousness and was accompanied by motor aphasia. His right limb muscle strength was level 5, and his left limb muscle strength was level 2 with sensory loss of the left limb. Brain CT showed a small amount of bleeding in the right basal ganglia (Fig. 3a). Brain magnetic resonance imaging (MRI) showed a new cerebral infarction in the bilateral basal ganglia and bilateral cortex (Fig. 3b). Pathological examination of bilateral thrombosis showed mixed thrombosis (Fig. 4). The patient has been followed up at the local hospital postoperatively and his mRS score was 2 at our 90-day telephone follow-up.

## Discussion

Acute bilateral occlusion of the MCA is rare, accompanied by a poor prognosis, a high disability rate and a high mortality rate, and is mainly characterized by bilateral paralysis, epilepsy, coma, sudden systemic ischemia related to decerebellar rigidity, and even death [7, 8]. To our knowledge, there are eight reports of MT of the acute bilateral MCA or the bilateral internal carotid artery system in the literature (Table 1). Dietrich et al. described a case of sequential thrombectomy of the M1 segment of the MCA using suction and a stent in 2014 with a good prognosis [9]. Subsequently, Pop et al. reported two cases of bilateral thrombosis of the MCA using stents alone in the same year, but their prognoses were completely different [10]. In 2018, Braksick et al. also reported a case of hemiplegia and dysarthria at the beginning of the disease [11]. Brain CT showed a high-density shadow of the right MCA. The patients were unconscious shortly after intravenous alteplase thrombolytic therapy. Cerebral CTA examination revealed bilateral occlusion of the MCA and emergency embolectomy. However, the postoperative recovery of the patient was poor. Furthermore, in 2019, Larrew et al. described a method of bilateral internal carotid artery occlusion and simultaneous thrombectomy by two interventional teams, but the therapeutic outcome was fatal [12]. Storey et al. successfully performed sequential MT of the M1—M2 MCA using a combination of a stent thrombectomy device and suction technique, and a only slight neurological deficits were left [13]. London et al. reported a case of bilateral occlusion of the MCA in the M1 segment [14]. Emergency thrombectomy of the bilateral MCA was performed after IVT was ineffective. The patient eventually did not have sequelae. Jeromel et al. also reported a case of aggravated symptoms during IVT [15]. Imaging examination showed bilateral occlusion of the MCA, but the patient was finally treated with unilateral embolization of the MCA. To our knowledge, we report the first case of acute occlusion of the M1 segment of the unilateral MCA at admission and sudden aggravation of symptoms during IVT. Bilateral MCA occlusion of the M1 segment was found during the bridge, and a successful thrombectomy was performed in patients with good prognosis.

**Table 1:** Comparing 8 reported cases of MT in acute bilateral MCA and/or ICA occlusions

No	Author(s), year	Age, sex	Presentation	Occlusion location		Management	Prognosis
				ICA	MCA		
1	Dietrich et al. 2014 [9]	72/M	Left hemiparesis, progressing to coma	-	+ (M1)	aspiration + stent-retriever	minor deficit
2	Pop et al. 2014 [10]	78/F	Impaired consciousness	+	+ (M2)	stent-retriever	no deficit
3	Pop et al. 2014 [10]]	66/F	right sided weakness	+	+ (M1)	stent-retriever	severe deficit
4	Braksick et al. 2018 [11]	76/F	coma	-	+ (M1)	- (no data)	coma
5	Larrew et al. 2019 [12]	-	coma	+	+ -	aspiration	fatal
6	Storey et al. 2019 [13]	64/F	hemiparesis/hemiplegia	-	+ (M1,M2)	aspiration + stent-retriever	minor deficit
7	London et al. 2019 [14]	84/F	Aphasia/faciobrachial palsy/seizures	-	+ (M1,M2)	aspiration + stent-retriever	no deficit
8	Jeromel et al*.* 2019 [15]	77/F	Hemiplegia/dysarthri, progressing to coma	+	+ (M1,M2)	aspiration	severe deficits

Bilateral carotid occlusion may be caused by atherosclerotic thrombosis, dissection, or cardiac embolism. Bilateral occlusion of the MCA is usually caused by cardiogenic disease, such as atrial fibrillation with or without an atrial thrombus. Cardiogenic cerebral infarction has been reported to have a higher probability of recanalization than other cerebral infarctions after thrombolytic therapy because of the embolus components [16]. Moreover, the patient's onset time was within 3 h, so we first administered IVT with alteplase. In the process of IVT, the patient suddenly had unclear consciousness. After excluding secondary intracranial hemorrhage, we considered that there may be another embolism event. The patient had a history of paroxysmal atrial fibrillation, and atrial fibrillation can lead to left atrial and left atrial appendage thrombosis. Boris et al. found that the fresh thrombus in the atrial appendage was composed of a large number of fibrinogen and rich platelets through autopsy experiments in 76 patients with cardiogenic embolism, so the thrombus in the left atrial appendage was found to be more sensitive to thrombolytic drugs than other parts [17]. The patient's embolus is characterized by mixed thrombosis and supports embolus originating from the heart. Therefore, we considered patients in whom alteplase IVT leads to left atrial or left atrial appendage thrombus falling off the left ventricle, and then the thrombus from the left ventricle through the artery to the left MCA leads to embolism events.

The significance of this case report shows that patients with atrial fibrillation can present with multiple embolization times occurring in a relatively short period of time.

When performing an emergency MT procedure, we recommend that all intracranial angiograms be completed before embolization of the vessel responsible for the lesion if the condition permits.

## Conclusion

In conclusion, acute bilateral occlusion of the M1 segment is extremely rare and is accompanied by high morbidity and high mortality. Intravenous alteplase thrombolysis may increase the risk of shedding of the atrial thrombus in patients with atrial fibrillation. Nevertheless, early endovascular treatment can decrease the morbidity and mortality of such patients.

## Data Availability

The datasets generated during and/or analyzed during the current study are available from the corresponding author on reasonable request.

## Abbreviations

CT: Computed tomography
CTA: Computed tomography angiography
MCA: Middle cerebral artery
Tici: Thrombolysis in cerebral infarction
MT: Mechanical thrombectomy
IVT: Intravenous thrombolysis
